# Neuropeptides Function in a Homeostatic Manner to Modulate Excitation-Inhibition Imbalance in *C. elegans*

**DOI:** 10.1371/journal.pgen.1003472

**Published:** 2013-05-02

**Authors:** Tamara M. Stawicki, Seika Takayanagi-Kiya, Keming Zhou, Yishi Jin

**Affiliations:** 1 Division of Biological Sciences, Section of Neurobiology, University of California San Diego, La Jolla, California, United States of America; 2 Howard Hughes Medical Institute, University of California San Diego, La Jolla, California, United States of America; Stanford University School of Medicine, United States of America

## Abstract

Neuropeptides play crucial roles in modulating neuronal networks, including changing intrinsic properties of neurons and synaptic efficacy. We previously reported a *Caenorhabditis elegans* mutant, *acr-2(gf)*, that displays spontaneous convulsions as the result of a gain-of-function mutation in a neuronal nicotinic acetylcholine receptor subunit. The ACR-2 channel is expressed in the cholinergic motor neurons, and *acr-2(gf)* causes cholinergic overexcitation accompanied by reduced GABAergic inhibition in the locomotor circuit. Here we show that neuropeptides play a homeostatic role that compensates for this excitation-inhibition imbalance in the locomotor circuit. Loss of function in genes required for neuropeptide processing or release of dense core vesicles specifically modulate the convulsion frequency of *acr-2(gf)*. The proprotein convertase EGL-3 is required in the cholinergic motor neurons to restrain convulsions. Electrophysiological recordings of neuromuscular junctions show that loss of *egl-3* in *acr-2(gf)* causes a further reduction of GABAergic inhibition. We identify two neuropeptide encoding genes, *flp-1* and *flp-18*, that together counteract the excitation-inhibition imbalance in *acr-2(gf)* mutants. We further find that *acr-2(gf)* causes an increased expression of *flp-18* in the ventral cord cholinergic motor neurons and that overexpression of *flp-18* reduces the convulsion of *acr-2(gf)* mutants. The effects of these peptides are in part mediated by two G-protein coupled receptors, NPR-1 and NPR-5. Our data suggest that the chronic overexcitation of the cholinergic motor neurons imposed by *acr-2(gf)* leads to an increased production of FMRFamide neuropeptides, which act to decrease the activity level of the locomotor circuit, thereby homeostatically modulating the excitation and inhibition imbalance.

## Author Summary

Imbalanced neuronal circuit activity is considered a major underlying cause in many neurological disorders, such as epilepsy and autism. Neuropeptides are small polypeptides that are released from neurons. They are widely known to provide neuromodulatory functions and have diverse roles in the nervous system. By investigating a *C. elegans* mutant that exhibits convulsions as the result of an imbalanced excitation and inhibition in the locomotor circuit, we have identified a homeostatic mechanism involving two distinct neuropeptide genes. We find that the expression of the neuropeptides is up-regulated in response to over-excitation and that, in turn, they act to increase inhibitory transmission. While current treatment strategies for epilepsy have focused on targeting fast synaptic transmission, this work supports the general notion that manipulating slow neuropeptide neurotransmission can strongly influence neural excitation and inhibition imbalance.

## Introduction

Neuropeptides are widespread and diverse modulators of neuronal circuit function, and have long been known to play regulatory roles in complex behaviors, such as learning, feeding, temperature regulation, and pain sensation [1], [2]. Additionally, neuropeptide modulation is implicated in a number of neurological diseases including epilepsy and autism [3]–[8]. In recent years great strides have been made in the recognition of the diverse means by which neuropeptides regulate neuronal circuits [9]–[16]. In particular, numerous studies from *C. elegans* have revealed important insights on the precise mechanisms underlying endogenous neuropeptide function in animal behaviors [15], [17]–[19].

The *C. elegans* genome contains over 100 peptide-encoding genes, which are generally classified as *flp* for FMRFamide-like peptides, *ins* for insulin-like genes, and *nlp* for neuropeptide-like proteins [20]. Recent proteomic studies have detected expression of over 150 distinct mature peptides [20]–[23]. As in higher vertebrates and other organisms, neuropeptide precursors are packaged into large dense core vesicles, and are further processed into functionally mature neuropeptides through a series of conserved enzymatic reactions [24], [25]. The release of dense core vesicles occurs in response to Ca^2+^ influx, and relies on several unique proteins in addition to those that are also involved in fast neurotransmitter release [26].

The two best characterized enzymes for neuropeptide processing in *C. elegans* are the proprotein convertase (PC2), EGL-3, and the carboxypeptidase E (CPE), EGL-21 [20], [27], [28]. EGL-3/PC2 cleaves the propeptide after the basic amino acid residues located at the C-terminus of the individual peptides [20]. EGL-21/CPE then removes the basic amino acids of the newly cleaved peptides [20]. Both genes are expressed primarily in the nervous system [20], [27], [28]. An early report using an antibody that recognizes fully processed FMRFamide-related peptides showed loss of most staining in *egl-21* mutants, and a great reduction of staining in *egl-3* mutants [27]. Recent peptidomic analyses fail to detect any processed neuropeptides in *egl-3* null mutants [21]. While *egl-21* mutants show incomplete processing of the majority of FLP and NLP peptides, they also express a number of fully processed peptides [22]. Thus, these two enzymes are important for the processing and production of most, but not all, mature neuropeptides. *egl-3* and *egl-21* mutants share similar phenotypes including retention of eggs, sluggish movement, and a reduction in sensitivity to the acetylcholinesterase inhibitor aldicarb [27], [28]. However, *egl-3; egl-21* double mutants show increased resistance to aldicarb, compared to either single mutant [27], [28], suggesting that they may not act in a completely linear pathway.

Neuropeptide release in *C. elegans* is well known to influence neural circuit activity and behavior [15], [29], [30]. The UNC-31 CAPS (Calcium-dependent Activator Protein for Secretion) protein is essential for peptide-containing dense core vesicle release, and *unc-31* mutants exhibit many sensory deficits and impaired locomotion [31]–[35]. Examples of specific neuropeptides regulating the locomotor circuit activity include the neuropeptide NLP-12, which is released by the stretch sensitive neuron DVA and can influence cholinergic motor neuron neurotransmitter release [14]. The levels of the FLP-1 FMRFamide peptides can also alter locomotor behavior such that *flp-1(lf)* mutants are hyperactive while overexpression of *flp-1* causes reduced mobility [36].

*C. elegans* sinusoidal locomotion is the result of coordinated muscle contraction due to innervation by the excitatory cholinergic motor neurons and inhibitory GABAergic motor neurons in the ventral cord [37]. Neuropeptide signaling has been implicated in modulating the activity of both types of motor neurons as well as the muscles [12], [20]. We have previously reported that the ACR-2 nicotinic acetylcholine receptor is expressed in the cholinergic motor neurons and plays a key role in balancing excitatory and inhibitory neurotransmission in the locomotor circuit [38]. Specifically, a gain of function mutation (Val309Met), designated as *acr-2(gf)*, in the pore-lining transmembrane domain of the ACR-2 subunit causes an increase in cholinergic excitation, accompanied with a decrease in GABAergic inhibition. This imbalance in excitation and inhibition results in stochastic convulsive behavior due to spontaneous contractions of body muscles. Thus, the frequency of convulsions of the *acr-2(gf)* mutant can be used as an indicator for the imbalanced activity of the locomotor circuit.

In this study we examined the roles of neuropeptides in modulating excitation and inhibition imbalance in the locomotor circuit. We show that neuropeptides processed by EGL-3 and released from the cholinergic motor neurons inhibit the convulsions caused by *acr-2(gf)*. We find that two neuropeptide-encoding genes, *flp-1* and *flp-18*, act together to reduce excitation and inhibition imbalance in the locomotor circuit. *acr-2(gf)* causes a specific up-regulation of *flp-18* expression in the cholinergic motor neurons. Electrophysiological recordings of the neuromuscular junctions indicate that *egl-3* and *flp* genes primarily influence GABAergic synaptic transmission. We also identify two neuropeptide receptors, NPR-1 and NPR-5 that are likely involved in the regulation of convulsions by the FLP-18 neuropeptides. These data suggest that neuropeptide production is regulated by activity, and that in turn neuropeptides function in a homeostatic manner to modulate output of the locomotor circuit. Our findings have implications for our understanding of excitation-inhibition imbalance in disease conditions, and support a general notion that neuropeptide modulation can provide effective strategies in disease management.

## Results

### Loss of function in the proprotein convertase EGL-3 increases the convulsion frequency of *acr-2(gf)*

**Figure 1:**
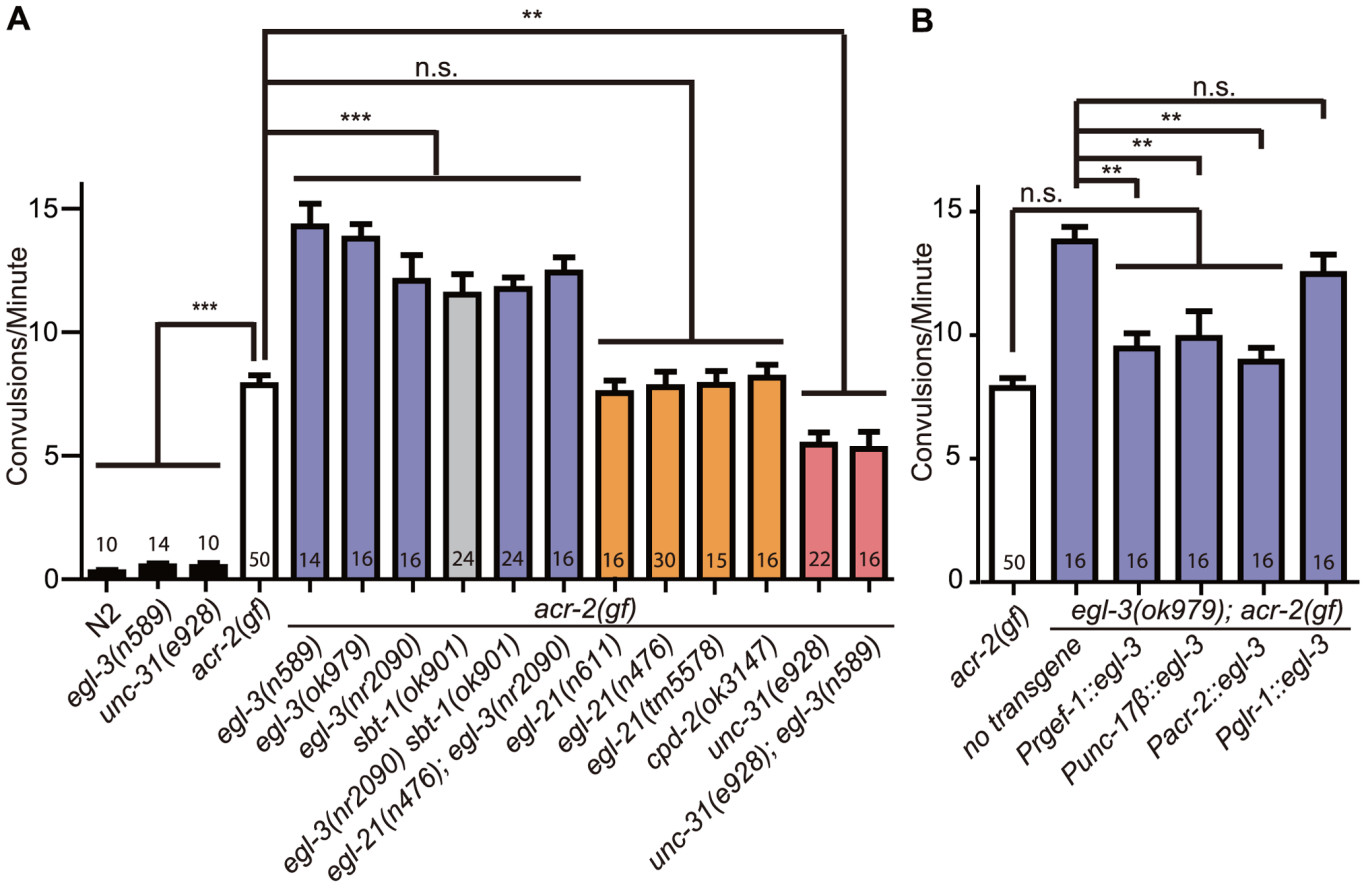
Neuropeptide processing and release pathway regulate *acr-2(gf)* convulsions. All mutations are loss of function alleles, except for *acr-2(gf)*, which designates *acr-2(n2420)*. Mean convulsion frequencies are shown. Error bars indicate SEM. Numbers in the graph indicate sample sizes. Statistics: ***: *p*<0.001, **: *p*<0.01, *: *p*<0.05 by ANOVA and Bonferroni post hoc test. (A) Loss of function in *egl-3* and *sbt-1* significantly enhances *acr-2(gf)* convulsions; and the increased convulsion caused by egl-3(lf) is dependent on *unc-31*. (B) *egl-3* functions in the cholinergic motor neurons to suppress *acr-2(gf)* convulsions. The number of independent transgenic lines tested are the following: *Prgef-1::egl-3*; 4 lines, *Punc-17β::egl-3*; 3 lines, *Pglr-1::egl-*3; 3 lines, *Pacr-2*; 2 lines. Quantification data is shown for one representative line.

To specifically test the roles of neuropeptides on *acr-2(gf)* induced convulsions, we first examined a set of mutants that are known to disrupt peptide processing. We found that multiple alleles of *egl-3* caused a significant increase in the convulsion frequency of *acr-2(gf)* (Figure 1A). A null mutation in *sbt-1*, a molecular chaperone necessary for EGL-3 function [23], showed a similar enhancement. *egl-3(lf) sbt-1(lf); acr-2(gf)* triple mutants showed a similar level of increased convulsions as *egl-3(lf); acr-2(gf)* and *sbt-1(lf); acr-2(gf)* double mutants, consistent with SBT-1 and EGL-3 acting in the same pathway. The overall locomotion pattern and speed of *sbt-1; acr-2(gf)* was indistinguishable from that of *acr-2(gf)* (Videos S1, S2), supporting the specific effects of SBT-1 and EGL-3 on convulsion frequency.

The carboxypeptidase E EGL-21 generally functions together with EGL-3 in producing mature neuropeptides [20]. However, we tested three mutations in *egl-21*, including a large deletion *tm5578*, which removes most of the exons 2 and 3 and causes premature stop after 85 amino acids (Table S1), and did not observe any effects on *acr-2(gf)* convulsions (Figure 1A). A null mutation in *cpd-2*, another carboxypeptidase, also showed no effects. Moreover, *egl-21(lf); egl-3(lf); acr-2(gf)* triple mutants behaved similarly to *egl-3(lf); acr-2(gf)*. These observations suggest that *egl-21* may not be required, or has a partial role, for processing the specific neuropeptides involved in *acr-2(gf)* convulsive behavior. As addressed later, we found the latter interpretation to be true. Overall, these observations indicate that EGL-3-dependent neuropeptides modulate the convulsive behavior of *acr-2(gf)* animals.

The function of neuropeptides is dependent on dense core vesicle release that requires the CAPS protein UNC-31 [26]. To test further the role of neuropeptides in modulating *acr-2(gf)* convulsions, we introduced a null mutation of *unc-31* into the *acr-2(gf)* background. In contrast to *egl-3(lf); acr-2(gf), unc-31(lf); acr-2(gf)* double mutants showed a significant reduction in the convulsion frequency as compared to the *acr-2(gf)* mutants alone (Figure 1A, Videos S1, S3). Importantly, *unc-31(lf)* blocked the enhancement of *egl-3(lf)*, as *egl-3(lf); unc-31(lf); acr-2(gf)* triple mutants convulsed to the same degree as *unc-31(lf); acr-2(gf)* (Figure 1A). Dense core vesicles contain complex components that include neuropeptides, whose processing most likely depends on EGL-3, as well as INS-like peptides, whose processing generally does not depend on EGL-3. Upon release, peptides can act in a combinatorial manner to modulate specific pathways. The fact that the enhanced convulsion in *egl-3(lf); acr-2(gf)* is dependent on *unc-31* led us to propose that the effective mature neuropeptides processed by EGL-3 are a specific subset of dense core vesicle components released via UNC-31.

We further addressed in which cells neuropeptide processing by EGL-3 is required to modulate *acr-2(gf)*. We found that expression of *egl-3(+)*, either pan-neuronally using the *rgef-1* promoter [39], or in the cholinergic motor neurons using the *unc-17β* or the *acr-2* promoter [32], fully rescued the enhanced convulsions in *egl-3(lf); acr-2(gf)*, whereas expression of *egl-3(+)* in pre-motor command neurons, driven by the *glr-1* promoter [40], did not show any effect (Figure 1B, Tables S1, S2). Together, these data reveal that neuropeptides processed in the cholinergic motor neurons modulate the convulsive behavior of *acr-2(gf)*, and suggest that the neuropeptide products act to restore the balance of excitation and inhibition in the locomotor circuit.

### FMRFamide-like peptides encoded by *flp-1* and *flp-18* act synergistically to decrease locomotor circuit activity in *acr-2(gf)* mutants

**Figure 2:**
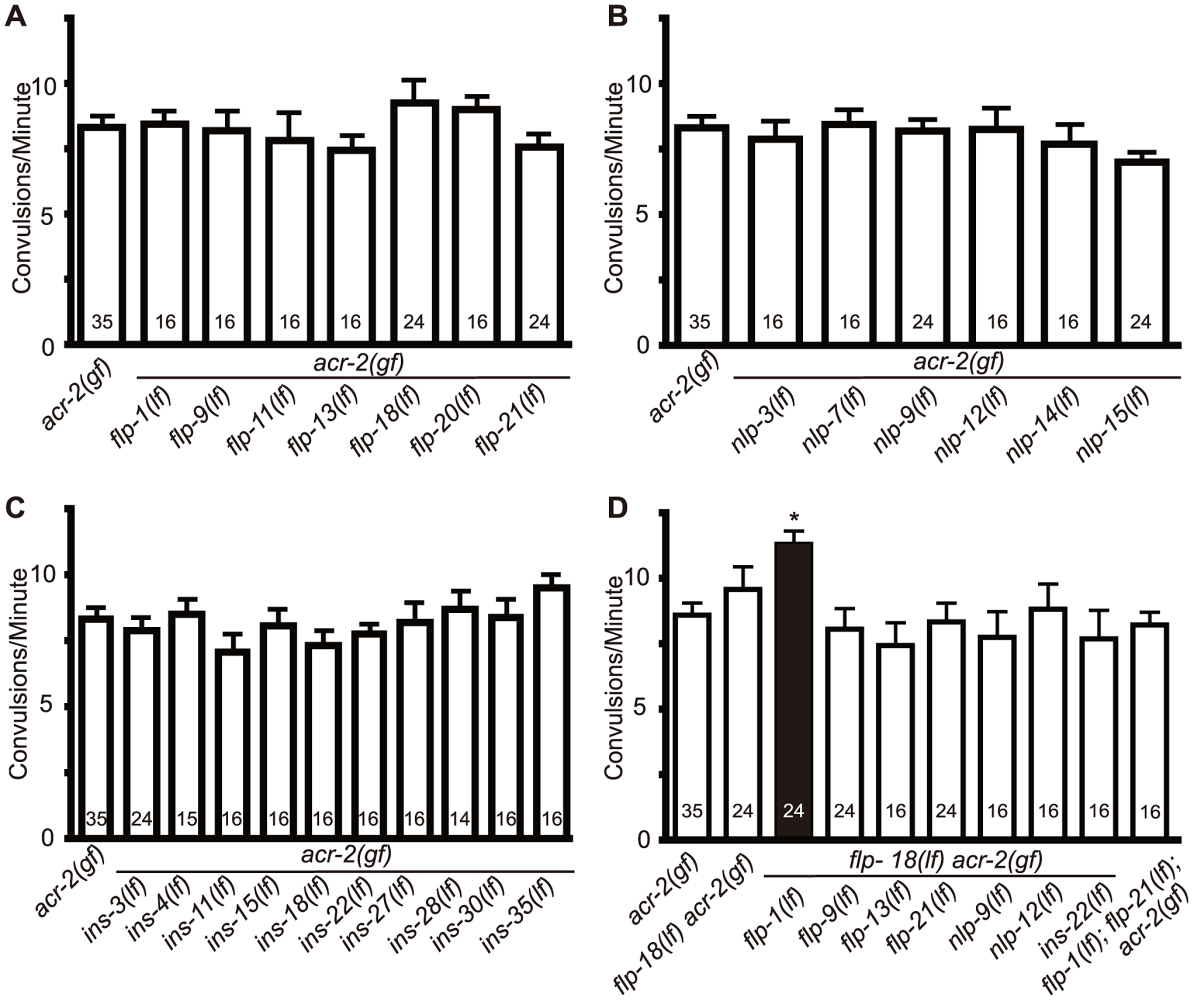
Loss of both *flp-1* and *flp-18* enhances *acr-2(gf)* convulsions. Null mutants of candidate neuropeptide genes were tested for effects on *acr-2(gf)* convulsions. *flp-18(lf)* indicates *flp-18(tm2179)*; the allele number for other genes are listed in materials and methods. No significant effects were observed for selected FMRF-amide (*flp*) (A), neuropeptide like proteins (*nls*) (B), or insulins (*ins*) (C). (D) Double mutants of candidate peptide genes with *flp-18*. Loss of both *flp-1* and *flp-18* leads to a significant enhancement of acr-2(gf) convulsions. Numbers in the graph indicate sample sizes. Mean convulsion frequencies are shown. Error bars indicate SEM. Statistics: *: *p*<0.05 by ANOVA and Dunnett's post hoc test.

**Figure 3:**
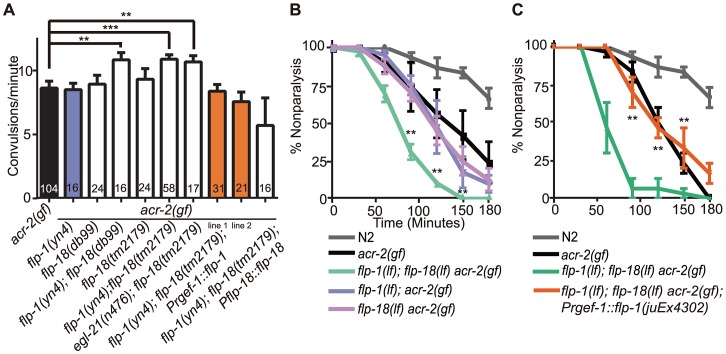
*flp-1* and *flp-18* act as inhibitory neuropeptides in the *acr-2(gf)* background. (A) Convulsion frequency of *acr-2(gf)* in combination with loss of function (lf) mutations in *flp-1(yn4)*, *flp-18(tm2179)*, or *flp-18(db99)*. The enhanced convulsion frequency of *flp-1(lf); flp-18(lf) acr-2(gf)* animals is rescued with transgenic expression of *flp-1* under the pan-neuronal promoter *Prgef-1*. Two independent transgenic lines were tested as indicated by line 1 and 2. Mean convulsion frequencies are shown. Error bars indicate SEM. Numbers in the graph indicate sample sizes. Statistics: ***: *p*<0.001, **: *p*<0.01 by ANOVA and Dunnett's post-hoc test. (B, C) Rate of paralysis on 150 µM aldicarb plates in *acr-2(gf)* background. *flp-1(lf); flp-18(lf)* mutants in the *acr-2(gf)* background showed enhanced aldicarb sensitivity compared to *acr-2(gf)* (B). Pan-neuronal expression of *flp-1* rescues the increased aldicarb sensitivity of the *flp-1(lf); flp-18(lf) acr-2(gf)* mutants (C). n = 10 for one group per trial; and results of three to five independent trials are shown. Mean rate of paralysis are shown for each time point. Error bars indicate SEM. Two independent transgenic lines were tested, only one is shown in the graph. Statistics in B, C: **: *p*<0.01, *: *p*<0.05 by two-way ANOVA and Bonferroni post-hoc test.

We next sought to determine the specific neuropeptides responsible for the inhibition of *acr-2(gf)* convulsions. We tested a set of candidate neuropeptide genes that had either been shown to be expressed in the locomotor circuit, or were known to affect locomotion [20]. Of 23 neuropeptide mutants tested, none showed significant enhancement of the *acr-2(gf)* convulsion phenotype (Figure 2A–2C, Table S1). We reasoned that the observed inhibitory effects of *egl-3* could be due to a group of neuropeptides produced by more than one gene. To test this idea, we made selected double mutants among *flp* and *nlp* genes chosen based on similarity in expression patterns or phenotypes. In doing so we found that eliminating both *flp-1* and *flp-18* resulted in a significant enhancement of *acr-2(gf)* convulsions (Figure 2D, Figure 3A). Two independent *flp-18(lf)* mutants, *flp-18(tm2179)* and *flp-18(db99)*, gave similar effects (Figure 3A). None of the other seven neuropeptide gene double mutants affected *acr-2(gf)* convulsion frequency (Figure 2D). We note that while *flp-1; flp-18 acr-2(gf)* mutants display a significant enhancement of the convulsion frequency, the extent of the convulsion is often less obvious than that seen in *egl-3(lf); acr-2(gf)* animals (Videos S4, S5), suggesting that other as yet unidentified neuropeptides may also be influencing *acr-2(gf)*.

In the recent peptidomic studies of *egl-21(lf)* animals, fully processed FLP-1 peptides are reported to be largely undetectable; however, four of the six fully processed mature peptides from FLP-18 are produced [22]. The presence of functional FLP-18-derived peptides would explain why *egl-21(lf)* single mutants did not show detectable effects on *acr-2(gf)* (Figure 1A). To test this idea, we constructed *egl-21(lf); flp-18(lf) acr-2(gf)* triple mutants, and observed that the convulsion frequency in these animals was comparable to that of *flp-1(lf); flp-18(lf) acr-2(gf)* (Figure 3A). Thus, these observations support a role of EGL-21 in the processing of FLP-1 neuropeptides, and imply other unidentified carboxypeptidases in the processing of FLP-18 neuropeptides.

As an independent assay for the effects of *flp-1* and *flp-18* neuropeptides on the locomotor circuit activity associated with *acr-2(gf)* convulsions, we tested the sensitivity of animals to the acetylcholinesterase inhibitor aldicarb [41]. *acr-2(gf)* animals show hypersensitivity to aldicarb, consistent with increased cholinergic transmission and decreased GABAergic transmission [38] (Figure 3B, 3C). *flp-1(lf)* mutants showed mild resistance to aldicarb (Figure S1A), consistent with a previous report [29]. *flp-18(lf)* showed sensitivity to aldicarb similar to wild type and suppressed the resistance of *flp-1(lf)* (Figure S1A). The hypersensitivity of *acr-2(gf)* to aldicarb was slightly, but not significantly enhanced by loss of function mutations in either *flp-1* or *flp-18* alone (Figure 3B). Notably, triple mutants of *flp-1(lf); flp-18(lf) acr-2(gf)* showed significantly increased sensitivity to aldicarb, compared to *acr-2(gf)* alone (Figure 3B). Both the increased convulsion frequency and the increased aldicarb sensitivity of the *flp-1(lf); flp-18(lf) acr-2(gf)* triple mutants were rescued by transgenic expression of *flp-18*, as well as pan-neuronal expression of *flp-1(+)* (Figure 3A, 3C), indicating FLP genes act in the nervous system to modulate the excitation-inhibition imbalance caused by *acr-2(gf)*.

### EGL-3 and FLP neuropeptides primarily regulate GABAergic inhibition

**Figure 4:**
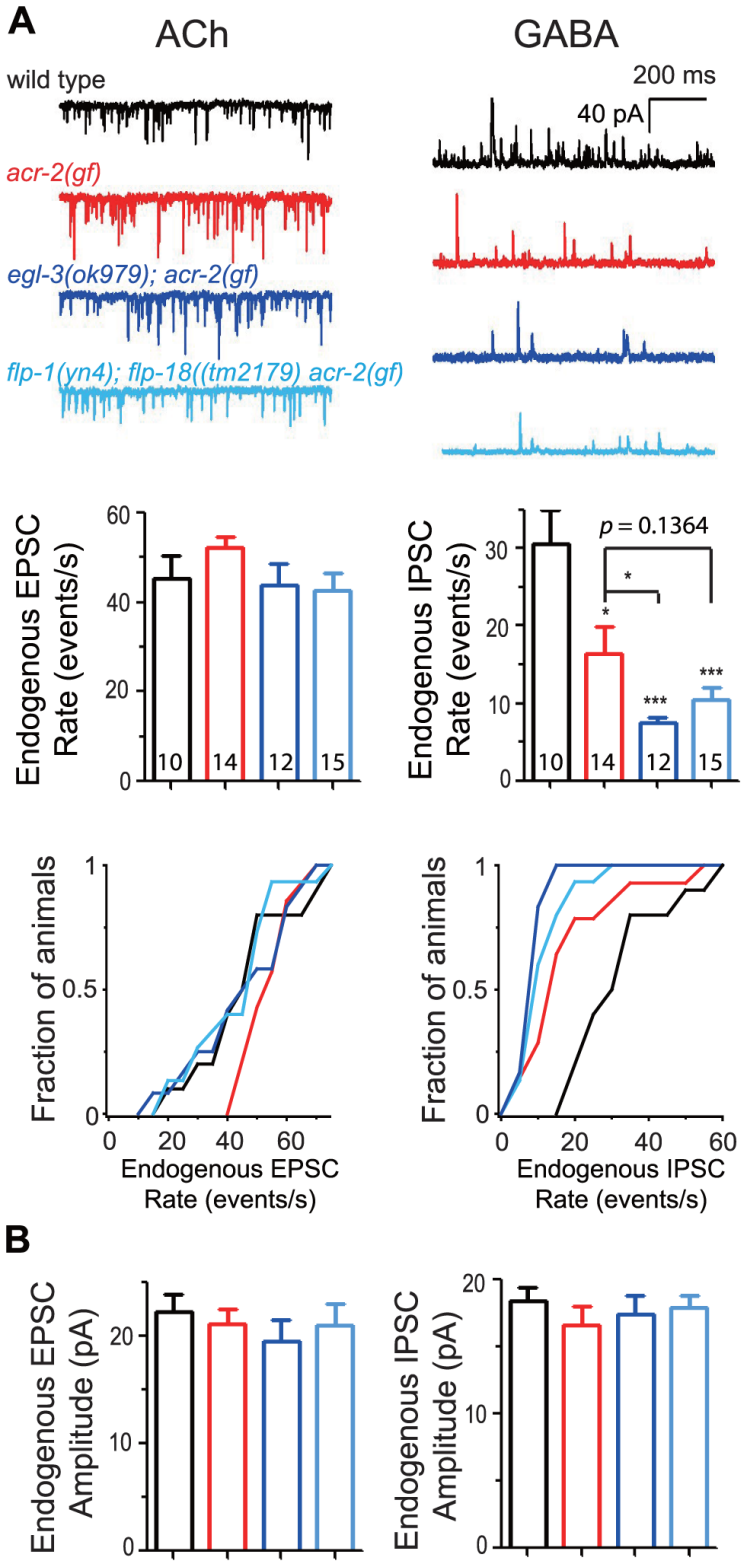
Neuropeptide modulation primarily affects GABAergic neuromuscular transmission. (A) Shown are the electrophysiology recording data on the neuromuscular junctions. Top panels are representative traces of genotypes indicated. Middle panels are mean rates of endogenous EPSCs and IPSCs; and bottom panels are cumulative fractions of the animal number with endogenous EPSC rate or IPSC rate less than indicated values in X-axis of genotype indicated. (B) Mean amplitudes of endogenous EPSCs and IPSCs from genotypes shown in A. The number of animals analyzed is indicated for each genotype. Error bars indicate SEM. Statistics, two-tailed t-test, ***, *p*<0.001; *, *p*<0.05.

To address more precisely how neuropeptides influence locomotor circuit activity in the *acr-2(gf)* background, we performed electrophysiological recordings at the neuromuscular junction. As reported previously [38], [42], when recordings were performed with 2 mM Ca^2+^ in the bath solution, *acr-2(gf)* showed slightly increased frequencies of endogenous acetylcholine release (EPSC), but a striking reduction of endogenous GABAergic activity (IPSC) (Figure 4A). Loss of *egl-3* function in *acr-2(gf)* caused a further reduction in endogenous IPSC frequency (Figure 4A). We observed a similar, but milder, effect on IPSC rate in *flp-1(lf); flp-18(lf) acr-2(gf)* triple mutants, consistent with the milder enhancement in convulsions in these animals (Videos S4, S5). *egl-3(lf); acr-2(gf)* and *flp-1(lf); flp-18(lf) acr-2(gf)* both showed slightly reduced endogenous EPSC rates compared to *acr-2(gf)* single mutant, although the average rate did not significantly differ among the strains (Figure 4A). The amplitudes of endogenous EPSCs and IPSCs were similar in all four genotypes tested (Figure 4B), suggesting that the muscle ACh and GABA receptors are largely unaltered. Thus, the electrophysiology analysis indicates that neuropeptides processed by EGL-3 compensate for the excitation-inhibition imbalance caused by *acr-2(gf)* primarily by influencing GABAergic transmission, and that FLP-1 and FLP-18 peptides account for most, but not all, of the neuromodulatory effects of EGL-3.

### The *acr-2(gf)* mutation increases FLP-18 expression in the cholinergic motor neurons

The specific effect of *flp-1* and *flp-18* on *acr-2(gf)* could be caused by either increased expression or release of these neuropeptides in this mutant background. To address the possibility of increased neuropeptide release, we examined two fluorescent reporters for dense core vesicle release from the cholinergic motor neurons: *Punc-129::NLP-21::venus* and *Punc-129::INS-22::venus* [29], [43]. Neither reporter showed significant changes in fluorescence intensity or pattern (Figure S2), suggesting that the general release machinery is largely normal in *acr-2(gf)*.

**Figure 5:**
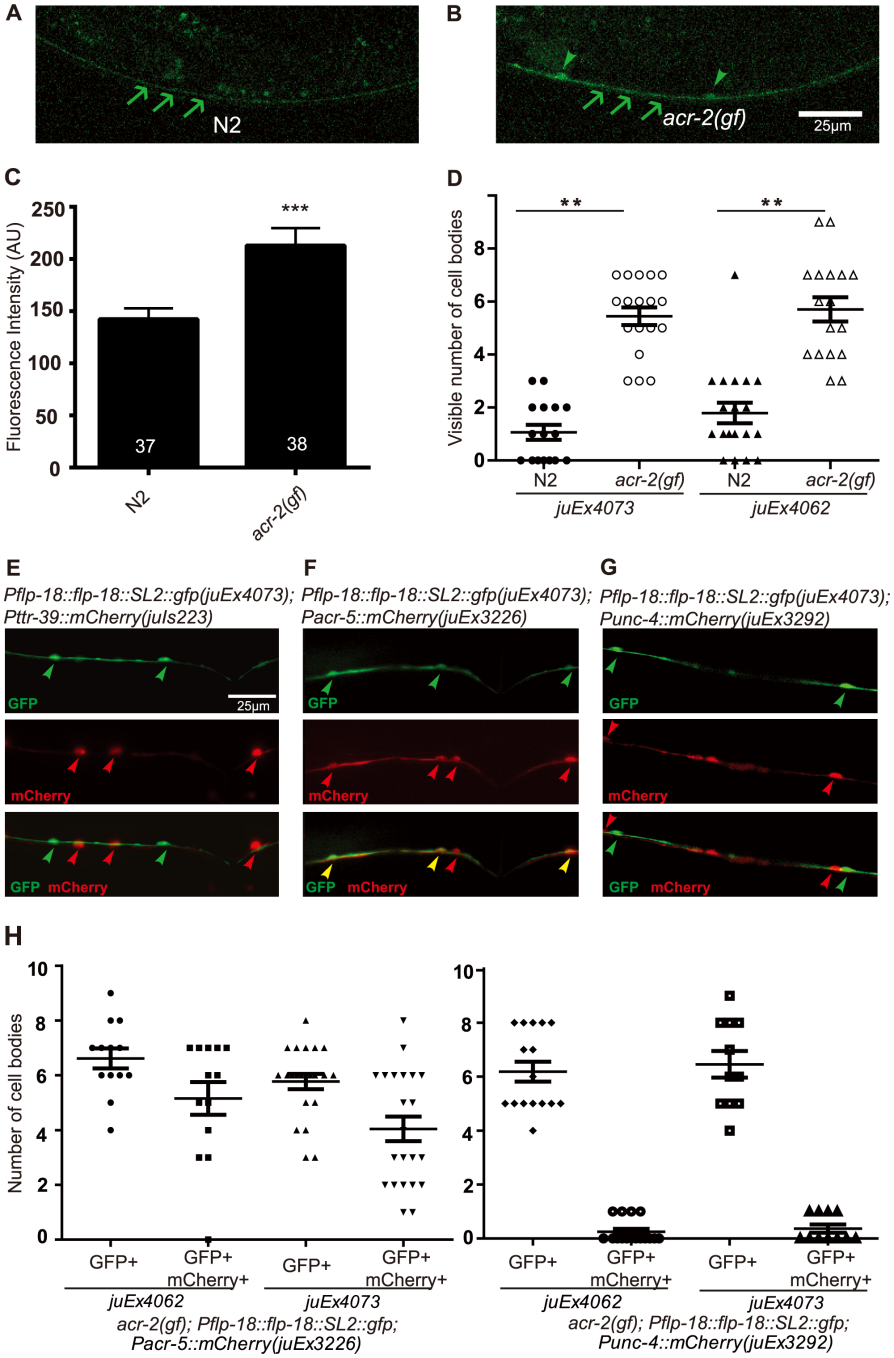
FLP-18 expression is selectively increased in the cholinergic motor neurons in the *acr-2(gf)* background. (A–B) Ventral nerve cord expression of *Pflp-18::flp-18::SL2::gfp* in wild type (N2) and *acr-2(gf)* background, respectively. Increased fluorescence intensity and cell body expression is seen in the ventral cord in the *acr-2(gf)* background. Arrows point to the ventral nerve cord posterior to the vulva, and arrowheads point to cell bodies. Scale bar = 25 µm. Two *Pflp-18::flp-18::SL2::gfp* transgenic lines, *juEx4062* and *juEx4073*, were tested. Images from *juEx4073* are shown. (C) Quantification of average fluorescence intensity in the ventral nerve cord posterior to the vulva. Mean fluorescence intensities are shown. N = 37 (wild type),  = 38 (*acr-2(gf)*). ***: *p*<0.001 by student's t-test. Error bars indicate SEM. (D) Quantification of the number of cell bodies in the ventral cord with visible GFP expression. ***: *p*<0.001, **: *p<*0.01 by Student's t-test. Each dot indicates quantification from one animal. Means are indicated by lines. Error bars indicate SEM. Two transgenic lines were tested. (E–H) Identification of the cells showing up-regulation of *Pflp-18::flp-18::SL2::gfp* in the *acr-2(gf)* background. (E) Co-expressing mCherry in GABAergic (*Pttr-39*) motor neurons did not show overlap with *Pflp-18::flp-18::SL2::gfp*. (F) Expression of mCherry in B-type (*Pacr-5*) cholinergic motor neurons overlapped extensively with *Pflp-18::flp-18:SL2::gfp* expression. (G) mCherry expression in A-type (*Punc-4*) cholinergic motor neurons mostly did not overlap with *Pflp-18::flp-18::SL2::gfp* expression. (H) Quantification of the number of cell bodies that showed overlapping expression of *Pflp-18::flp-18::SL2::gfp* and *Pacr-5::mCherry* or *Punc-4::mCherry* in F–G. Each dot indicates quantification from one animal. Means are indicated by lines. Error bars indicate SEM.

We next tested for increased expression of neuropeptides using a bicistronic *flp-18* reporter that contains the entire genomic locus of *flp-18*, including the 3.6 kb upstream promoter, followed by a trans-spliced SL2::GFP (designated as *Pflp-18::flp-18::SL2::gfp*) [44]. In the wild type background this reporter was strongly expressed in several head neurons and was detectable at low levels in the ventral nerve cord. In the *acr-2(gf)* background, we found that *Pflp-18::flp-18::SL2::gfp* expression in the ventral cord neurons was strongly enhanced (Figure 5A–5C), while its expression in the head neurons was not changed (Figure S3). GFP expression pattern in the cholinergic motor neurons under the *unc-17β* promoter was also not affected by *acr-2(gf)* mutation (Figure S4). We quantified the number of ventral cord neuron cell bodies that showed expression of *Pflp-18::flp-18::SL2::gfp*, and found that more cell bodies were observed with elevated expression in *acr-2(gf)* animals than in wild type (Figure 5D). We were not able to examine *flp-1* expression due to variable expression patterns of different transgenic *flp-1* reporter lines (our unpublished data).

The cells that showed up-regulation of *Pflp-18::flp-18::SL2::gfp* in *acr-2(gf)* were evenly spaced along the ventral nerve cord (Figure 5B). To determine in which class of motor neurons *Pflp-18::flp-18::SL2::gfp* expression was affected, we crossed *acr-2(gf); Pflp-18::flp-18::SL2::gfp* with a set of mCherry reporter lines driven by specific motor neuron promoters. We observed consistent co-expression of GFP and mCherry in B-type cholinergic motor neurons, labeled by *Pacr-5*, and occasional expression in A-type cholinergic motor neurons, labeled by *Punc-4*, but no overlapping expression in GABAergic D-type motor neurons, labeled by *Pttr-39* (Figure 5E–5H). These data indicate that *acr-2(gf)* primarily up-regulates *flp-18* expression in the cholinergic B-type motor neurons.

### Elevated *flp-18* expression correlates with the onset of convulsions and is likely induced by neuronal activity

**Figure 6:**
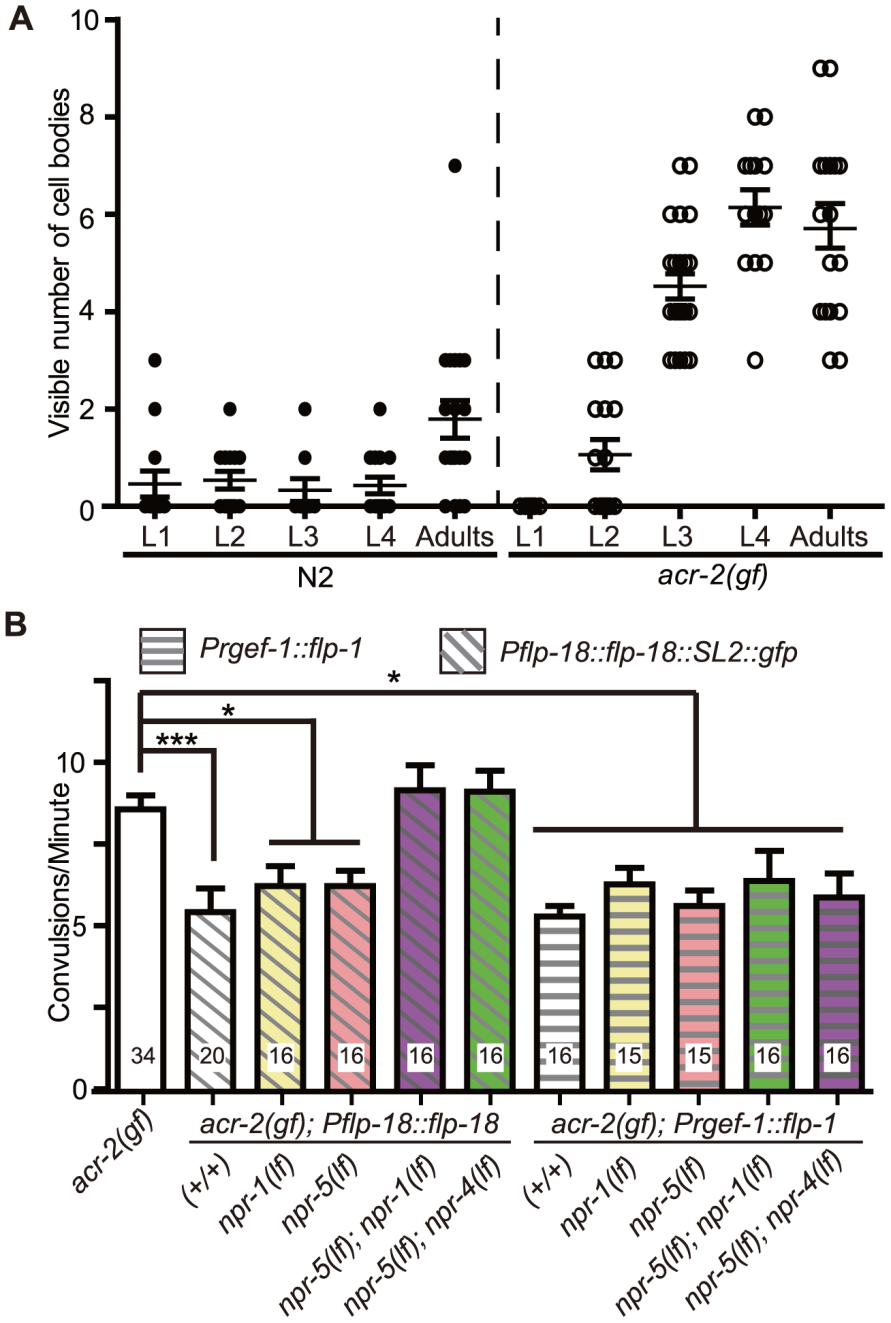
Induced expression of FLP-18 in *acr-2(gf)* correlates with the onset of convulsions, and high levels of FLP-18 or FLP-1 suppress convulsions. (A) Quantification of the number of cell bodies in the ventral cord that showed *Pflp-18::flp-18::SL2::gfp* expression in larval and adult stages. Each dot indicates quantification from one animal. Means are indicated by lines. Error bars indicate SEM. Two independent lines *juEx4062* and *juEx4073* were tested. Result from *juEx4073* is shown. (B) Convulsion of *acr-2(gf)* was suppressed by expression of *Pflp-18::flp-18::SL2::gfp* or pan-neuronal expression of *flp-1*. The suppression by *flp-18* overexpression was blocked by loss of both *npr-1* and *npr-5*, or *npr-4* and *npr-5*. The same set of *npr* mutations did not affect the suppression effect of *flp-1* overexpression. Mean convulsion frequencies are shown. Error bars indicate SEM. Statistics: ***: *p*<0.001, *: *p*<0.05 by ANOVA and Dunnett's post-hoc test. *(+/+)* indicates strains with no mutations in any of the neuropeptide receptor genes.

To further correlate the *acr-2(gf)*-dependent up-regulation of *flp-18* expression, we examined the developmental onset of *Pflp-18::flp-18::SL2::gfp* expression with respect to the onset of convulsions. We have shown earlier that the onset of convulsions in *acr-2(gf)* mutants occurs in mid-larval stage [38]. We found that in *acr-2(gf)* mutants the expression of the *flp-18* reporter also increased sharply in mid-larval stages (Figure 6A). The close temporal correlation between the onset of *acr-2(gf)* convulsions and that of *flp-18* up-regulation in cholinergic motor neurons is consistent with *flp-18* up-regulation being caused by increased cholinergic activity. Supporting this idea, we observed increased expression of *Pflp-18::flp-18::SL2::gfp* in wild type animals acutely treated with aldicarb (Figure S5, Protocol S1). In contrast, the expression of *Pflp-18::flp-18::SL2::gfp* in *acr-2(gf)* animals was decreased when the animals were grown on plates with the acetylcholine receptor antagonist mecamylamine (Figure S5), which suppresses the convulsion behavior as previously reported [38].

As FLP-18 functions together with FLP-1 to reduce *acr-2(gf)* convulsions (Figure 2D, Figure 3A), we hypothesized that the induced expression of *flp-18* could be a homeostatic response to the elevated cholinergic neuronal activity in *acr-2(gf)*. If so, overexpression of *flp-18(+)* or *flp-1(+)* should ameliorate the extent of convulsions. Indeed, overexpressing *flp-18* under the control of its endogenous promoter caused a significant suppression of convulsions (Figure 6B). Overexpression of *flp-1*, driven by a pan-neuronal promoter, also resulted in a similar suppression of convulsions (Figure 6B). Together, these observations support the conclusion that in the *acr-2(gf)* background where excitation and inhibition balance is impaired, increased expression of *flp-18*, and possibly of *flp-1*, acts as a homeostatic response to dampen imbalanced circuit activity.

### *npr-1* and *npr-5* appear to be the major receptors mediating the suppression of convulsions by FLP-1 and FLP-18

**Figure 7:**
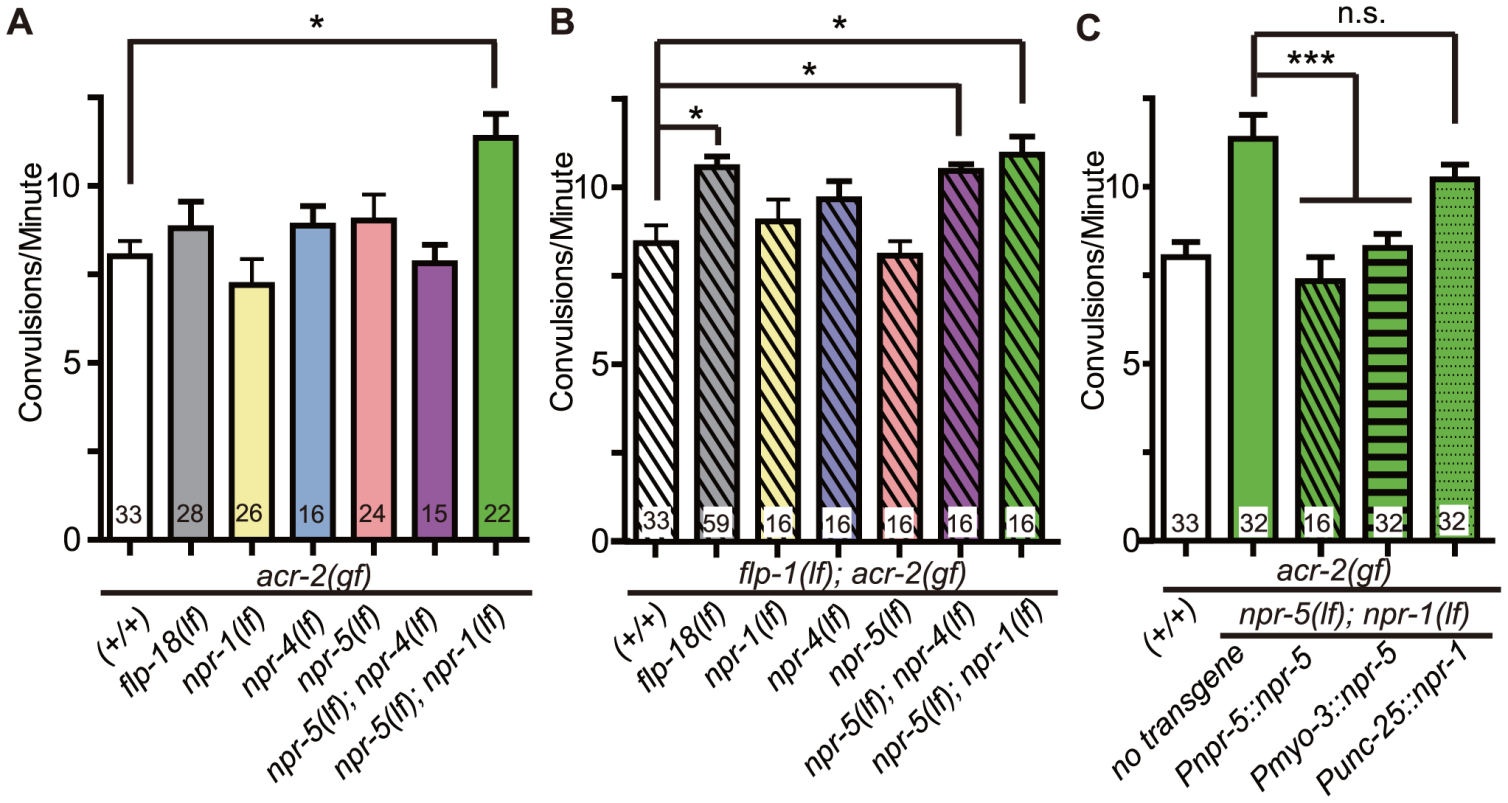
NPR-1, NPR-4, and NPR-5 act together to mediate the effects of neuropeptides on convulsions. (A–B). Convulsion frequencies of *acr-2(gf)* combined with loss of function mutations in *npr-1(ok1447)*, *npr-4(tm1782)*, *npr-5(ok1583)* (A) and with *flp-1(yn4)* (B). (C) Convulsion frequency of animals with cell type-specific expression of *npr-1* and *npr-5*. *npr-5* expression in the muscle rescued the increased convulsion frequency of *npr-5(lf); npr-1(lf) acr-2(gf)* triple mutant; two independent lines were tested. *npr-1* expression in GABAergic motor neurons did not significantly rescue the increased convulsion frequency; three lines were tested. All strains contain *acr-2(gf)*. Mean convulsion frequencies are shown. Error bars indicate SEM. Statistics: ***: *p*<0.001, *: *p*<0.05 by ANOVA and Bonferroni post hoc test. *(+/+)* indicates strain with no mutations in any of the neuropeptide receptor genes.

Neuropeptides generally act through G-protein coupled receptors (GPCRs). Next we sought to identify which GPCRs are involved in the regulation of convulsions by the *flp* neuropeptides. The CKR-2 receptor can be activated by FLP-1 at high concentration, and is also shown to act as a high-affinity receptor for NLP-12 [14], [45]. We found that *ckr-2(lf)* or *ckr-2(lf); flp-18(lf)* had no effects on *acr-2(gf)* (Figure S6), consistent with the observation that *nlp-12(lf)* did not affect *acr-2(gf)* either alone or in combination with *flp-18* (Figure 2D). Three receptors NPR-1, NPR-4 and NPR-5 can be activated by all six FLP-18 neuropeptides when expressed in *Xenopus* oocytes [44], [46]–[48]. NPR-1 is expressed in the ventral cord GABAergic motor neurons and in multiple head sensory neurons [46]. NPR-4 is expressed in the AVA, RIV, BDU and PQR neurons as well as in coelomocytes and the intestine [44]. NPR-5 expression is found in the amphid and phasmid neurons, interneurons AIA and AUA, as well as in the muscles [44]. We found that loss of function mutations in individual *npr* genes neither suppressed nor enhanced *acr-2(gf)* (Figure 7A). We then made selected double mutant combinations among *npr-1*, *npr-4*, and *npr-5*, in the presence or absence of *flp-1(lf)*. Eliminating both *npr-1* and *npr-5* in *acr-2(gf)* resulted in increased convulsions, while *npr-4(lf)* showed detectable effects only when both *npr-5* and *flp-1* were eliminated (Figure 7A, 7B). To further test the roles of these *npr* genes, we examined the suppression effects of *acr-2(gf)* by the overexpression of *flp-18*. We found that the suppression of convulsion by overexpression of *flp-18* were reduced by either *npr-1(lf); npr-5(lf)* or *npr-4(lf); npr-5(lf)* double mutations, but not by *npr-1(lf)* or *npr-5(lf)* single mutation (Figure 6B). Based on these observations, we conclude that NPR-1 and NPR-5 likely play a major role in mediating the modulatory action of FLP-18 in *acr-2(gf)*, while NPR-4 has a minor role. Similar *npr* receptor combinations had no effects on the suppression of convulsion by overexpression of *flp-1* driven by a pan-neuronal promoter (Figure 6B). It is possible that this reflects non-physiological effects caused by overexpression of *flp-1*. Alternatively, FLP-1 and FLP-18 may act through distinct signaling pathways. Supporting the latter idea, we observed a slight but significant difference in aldicarb sensitivity between *npr-5(lf); npr-1(lf) acr-2(gf)* and *flp-1(lf); npr-5(lf); npr-1(lf) acr-2(gf)* (Figure S7). Nonetheless, this difference did not result in significant changes in the convulsion frequency of *flp-1(lf); npr-5(lf); npr-1(lf) acr-2(gf)* from that of *npr-5(lf); npr-1(lf) acr-2(gf)* (Figure 7A, 7B), which could reflect the limitation of our visual detection methodology.

We next addressed the cell types in which *npr-1* or *npr-5* may act. We found that expression of *npr-5* in muscles by the *Pmyo-3* promoter reduced the convulsion frequency of *npr-5(lf); npr-1(lf) acr-2(gf)* animals to a similar degree as did *npr-5* expression under its endogenous promoter (Figure 7C). We also expressed *npr-1* in the GABAergic motor neurons by the *Punc-25* promoter, and did not detect any significant effect on the convulsion frequency of *npr-5(lf); npr-1(lf) acr-2(gf)*. Overall, our analysis supports a conclusion that NPR-5 acts in the muscle, while NPR-1 expressed in other neurons such as sensory head neurons may be contributing to the locomotor circuit activity in an indirect manner.

## Discussion

In this study we have identified two neuropeptide-encoding genes, *flp-1* and *flp-18* that act in a homeostatic manner to dampen the effects of the excitation-inhibition balance of the locomotor circuit caused by the *acr-2(gf)* mutation. The role of *flp-1* and *flp-18* in suppressing overexcitation of the locomotor circuit is dependent on neuropeptide processing by *egl-3* in the cholinergic motor neurons. We provide electrophysiological evidence that this neuropeptide modulation primarily acts on the GABAergic neural transmission at the neuromuscular junctions. Our previous studies have shown that *acr-2(gf)* elevates the activity of the cholinergic motor neurons [38]. Here, we find that *acr-2(gf)* causes up-regulation of *flp-18* expression in the cholinergic motor neurons, and that over-expression of *flp-18* or *flp-1* is able to suppress *acr-2(gf)*. Our analyses of known *flp* neuropeptide receptors suggest that *npr-1* and *npr-5* play a major role in mediating the peptide's function. We show that *npr-5* primarily acts in the muscles. Yet, the combinatorial effects of these receptors on the excitation-inhibition imbalance caused by *acr-2(gf)* likely involve multiple cell types.

The production of mature neuropeptides generally requires sequential enzymatic reactions starting with proprotein convertases, followed by carboxypeptidases [20]. Peptide mass spectrometry studies indicate that the majority of mature neuropeptides in *C. elegans* require EGL-3/PC2, its chaperone SBT-1, and EGL-21/CPE [20]. Previous studies have shown that *egl-3* and *egl-21* generally exhibit similar behavioral defects, although they differ in severity [27], [28]. Here, we find that loss of function in *egl-3* and *sbt-1*, but not in *egl-21*, enhances the convulsion frequency of *acr-2(gf)* animals. Proteomic analyses show that several mature peptides of FLP-18 are present in *egl-21* mutants [22]. We find that *egl-21(lf); flp-18(lf) acr-2(gf)* triple mutants show an increased convulsion frequency similar to *flp-1(lf); flp-18(lf) acr-2(gf)*. These data provide an explanation for the lack of effect on the *acr-2(gf)* convulsion frequency by the *egl-21* mutations, and also imply the involvement of other carboxypeptidases besides EGL-21 in mature neuropeptide production. Once mature peptides are processed in the dense core vesicles, the release of peptides requires UNC-31/CAPS. Intriguingly, we observed a suppression of *acr-2(gf)* convulsions by *unc-31(lf)*, an opposite effect from that of *egl-3(lf)*. *egl-3* is necessary for processing primarily NLP and FLP neuropeptides [21], [27], while *unc-31* is required for release of all neuropeptides including INS-like peptides and may also affect fast neurotransmitter release. We find that loss of both *flp-1* and *flp-18* largely mimicked the effects of *egl-3(lf)*. Importantly, the effects of *egl-3(lf)* are dependent on *unc-31(lf).* These data support a conclusion that *flp-1* and *flp-18* peptides, processed by EGL-3, are released via dense core vesicles and in turn act to modulate the locomotion circuit in an inhibitory manner. We infer that the suppression of convulsions of *acr-2(gf)* by *unc-31(lf)* is likely due to the involvement of other pathways or unidentified neuropeptides that play excitatory roles in locomotion.

Previous studies on *flp-18* have focused on the functions of FLP-18 released from the head interneurons [44]. *flp-18(lf)* mutants show defects in fat accumulation and foraging behavior, and the defects can be rescued by *flp-18* expression in the AIY or RIG neurons [44]. The role of FLP-18 neuropeptides in the locomotor circuit is unknown. In wild type animals, *flp-18* expression, visualized using a reporter that expresses FLP-18 and GFP under the endogenous *flp-18* promoter, is generally low in the cholinergic motor neurons [44] (Figure 5A). We find that the transcriptional expression of *flp-18* is specifically up-regulated in the cholinergic motor neurons at the onset of convulsions induced by *acr-2(gf)* and upon upregulation of cholinergic activity by acute aldicarb treatment. This result suggests that up-regulation of *flp-18* is likely a homeostatic response to the overexcitation caused by *acr-2(gf)*. We were not able to determine whether *flp-1* expression might be similarly regulated, due to inconsistent expression pattern of different transgenic reporter lines (our unpublished data, and C. Li, personal communication). Strong up-regulation of *flp-18* is consistently observed in the B type motor neurons, which drive forward locomotion [49], [50]. We have recently found that the AVB neurons, which provide major synaptic input to the B type neurons, are necessary for the onset of convulsions in *acr-2(gf)* [51]. Together, these observations support that the AVB-B neuron pathway plays a major role in the excitation-inhibition balance of the locomotor network.

FLP-18 can activate NPR-1, NPR-4 and NPR-5 receptors [44], [48]. Our analysis suggests that these receptors act in a combinatorial manner to modulate the convulsion in *acr-2(gf)* animals, with NPR-1 and NPR-5 having a major, and NPR-4 a minor role. These receptors are expressed in multiple cell types. Our data show that muscle-specific expression of NPR-5 can rescue the increased convulsions, suggesting that the FLP-18 neuropeptides can act directly on muscles to inhibit contraction, or to promote relaxation. NPR-1 and NPR-5 may also be activated by neuropeptides other than FLP-18, since *npr-5(lf); npr-1(lf) acr-2(gf)* triple mutants show a more severe phenotype than *flp-18(lf) acr-2(gf)* double mutants. The effect of *flp-1* and *flp-18* double loss of function is milder than that of *egl-3(lf)* (Figure 4), implying the involvement of other neuropeptides.

We previously showed that GABAergic transmission at the neuromuscular junctions is reduced in *acr-2(gf)* animals [38]. Our neuromuscular physiology analysis here shows that the neuropeptide modulation by *egl-3* and *flp-1* and *flp-18* primarily acts on GABAergic transmission. *npr-1* is expressed in the GABAergic motor neurons [44], [48]. However, our data suggests that this expression is unlikely to be directly responsible the effect of neuropeptides on GABA neurons. *flp-1* is reported to be expressed primarily in the head neurons including AIA, AIY, AVA, AVE, AVK, RIG, RMG, M5 [36]. The effects of *flp-1(lf)* on convulsions appear to be independent of CKR-2, presently the only known receptor for FLP-1 (Figure S6). *npr-1(lf); npr-5(lf)* double mutants cause enhanced convulsions of *acr-2(gf)*, similar to *flp-1(lf); flp-18(lf)* double mutants. Yet, double loss of function in *npr-1* and *npr-5* does not significantly affect the suppression of convulsions by *flp-1(+)* overexpression under a pan-neuronal promoter. It is possible that *flp-1(+)* overexpression activates other inhibitory pathways that do not require *npr-1* and *npr-5*. We observed an enhanced aldicarb sensitivity of the *flp-1(lf); npr-5(lf); npr-1(lf) acr-2(gf)* animals comparing to *npr-5(lf); npr-1(lf) acr-2(gf)* (Figure S7). However, this difference did not result in detectable differences in convulsion, which may likely be due to the limitation in our methodology of visual observation of convulsion. The modest effects of these receptors make it difficult to determine the precise contribution of their signaling in the context of convulsive behavior of *acr-2(gf)*. Identification of additional GPCRs that respond to FLP-1 will be necessary for fully understanding the peptidergic transmission pathway that modulates *acr-2(gf)* convulsions. Overall, our results are consistent with a model in which these neuropeptides act on multiple cell types, one of which is body wall muscle, to coordinate the activity state of the locomotion circuit.

The molecular nature and the physiological basis of *C. elegans acr-2(gf)* mutants share similarities with mutations causing epileptic seizures including an imbalance between excitation and inhibition of the nervous system. Examples of neuropeptides acting to inhibit altered neuronal circuit activity, such as in seizures, have also been observed in vertebrates. For example, the neuropeptide galanin has been shown to play a key role in epilepsy [5], [52]. Galanin agonists inhibit seizures [5], and expression of galanin is increased in the mouse brain upon the induction of seizures [53]. A model for the role of galanin in epilepsy has been proposed in that increased excitation increases galanin levels in an attempt to normalize the excitation and inhibition balance by reducing glutamatergic transmission [6]. Likewise, our studies have revealed that activity-dependent expression of neuropeptides provides a homeostatic mechanism to modulate neuronal network balance. Together, these findings provide support for manipulations of slow neuropeptide signaling in controlling neuronal circuit activity disruption underlying neurological disorders.

## Materials and Methods

### Genetics and alleles

All *C. elegans* strains were grown on NGM plates at room temperature (20–22°C) following standard methods. Deletion mutant strains were backcrossed two times against N2 before being used for strain construction. All double mutants were constructed using standard procedures, and genotypes were confirmed by PCR verification of the deletions. Table S1 lists the information on the alleles and strains. Specific alleles used in the figures are: *acr-2(gf)* indicates *acr-2(n2420), unc-31(e928)*, *egl-3(n589), egl-3(ok979), egl-3(nr2090), sbt-1(ok901), egl-21(n611), egl-21(n476), egl-21(tm5578), cpd-2(ok3147), flp-1(yn4), flp-9(ok2730), flp-11(tm2706), flp-13(tm2427), flp-18(tm2179), flp-20(ok2964), flp-21(ok889) ,nlp-3(tm3023), nlp-7(tm2984), nlp-9(tm3572), nlp-12(ok335), nlp-14(tm1880), nlp-15(ok1512), ins-3(ok2488), ins-4(ok3534), ins-11(tm1053), ins-18(ok3444), ins-22(ok3616), ins-27(ok2474)*, *ins-28(ok2722), ins-30(ok2343), ins-35(ok3297), npr-1(ok1447), npr-4(tm1782), npr-5(ok1583), ckr-2(tm3082), acr-2(ok1887).*

### Molecular biology and transgenes

Molecular biology was performed according to standard methods [54]. Expression constructs were generated using Gateway recombination technology (Invitrogen, CA), and Table S2 lists the details of the DNA clones generated in this study. An *unc-31* cDNA pDONR construct was provided by Dr. Kaveh Ashrafi [33], *Punc-17β::unc-31* was provided by Dr. Ken Miller [32], *Pglr-1::egl-3* , *Pacr-2::egl-3* and *Punc-25::npr-1* were provided by Dr. Josh Kaplan ([28] and personal communication), and *Pflp-18::flp-18::SL2::gfp* and *Pnpr-5::npr-5* were provided by Dr. Merav Cohen and Dr. Mario de Bono [44].

### Quantification of convulsion behavior

Ten to twenty L4 larvae were placed on freshly seeded NGM plates. The following day, young adults were transferred to fresh plates and recorded by video for 90 seconds, five frames per second. Eight animals were recorded for each genotype per trial and at least two trials were performed per genotype. Videos were scored by an observer blind to genotype. A “convulsion” was defined as a visible shortening in the animal's body length.

### Pharmacology analysis

L4 animals were picked the day before an experiment. The day of the experiment ten young adults per genotype were placed on plates containing 150 µM aldicarb, and the effects on animal movement were observed at 30 minute intervals. Animals were scored as paralyzed when no body movements were observed, even in response to touch.

### FLP-18 imaging

Confocal images were taken on a Zeiss LSM 510 with 1 µm per section, and processed using ImageJ. Maximum projection images were created from confocal stacks and the average intensity was measured of the ventral cord posterior to the vulva. For cell body counting, L4 animals were picked the day before an experiment, and young adults were observed using a Zeiss Axioplan 2 fluorescence microscope the following day. The number of cell bodies of the ventral nerve cord with visible GFP fluorescence was counted. For identification of the cells expressing *Pflp-18::flp-18::SL2::gfp* cell bodies with GFP and mCherry fluorescence were observed and counted. For the observation in different stages of animals, animals were synchronized at L1 stage and observed under Zeiss Axioplan 2 Fluorescence microscope at each developmental stage.

### Electrophysiology

NMJ dissection methods were adapted from previous studies [42]. In brief, adult worms were immobilized on Sylgard-coated cover slips with cyanoacrylate glue. A dorsolateral incision was made with a sharp glass pipette and the cuticle flap was folded back and glued down to expose the ventral medial body wall muscles. The preparation was then treated by collagenase type IV (Sigma-Aldrich) for ∼30 s at a concentration of 0.4 mg/ml. The bath solution contained (in mM): 127 NaCl, 5 KCl, 26 NaHCO_3_, 1.25 NaH_2_PO_4_, 2 CaCl_2_, 4 MgCl_2_, 10 glucose, and sucrose to 340 mOsm, bubbled with 5% CO_2_, 95% O_2_ at 20°C. The pipette solution containing (in mM): 120 CH_3_O_3_SCs, 4 CsCl, 15 CsF, 4 MgCl_2_, 5 EGTA, 0.25 CaCl_2_, 10 HEPES and 4 Na_2_ATP, adjusted to pH 7.2 with CsOH. Conventional whole-cell recordings from muscle cells were performed at 20°C with 2–3 MΩ pipettes. An EPC-10 patch-clamp amplifier was used together with the Patchmaster software package (HEKA Electronics, Lambrecht, Germany). Endogenous acetylcholine postsynaptic currents were recorded at −60 mV and GABA postsynaptic currents were recorded at 0 mV. The current traces were imported to IGOR Pro (WaveMetrics, Lake Oswego, OR) for further analysis.

### Ethics statement

This work does not use human subjects or animals. The research was performed following the ethical conduct rules of University of California San Diego.

## Supporting Information

Figure S1 Aldicarb sensitivity of *flp-1* and *flp-18* mutants. (A) in wild type and (B) in *acr-2(lf)* background. Animals were placed on an NGM plate with 1 mM (A) or 500 µM (B) aldicarb and non-paralyzed worms were counted every 30 minutes. (A) *flp-1(lf)* show aldicarb resistance. (B) Loss of *flp-1* and *flp-18* does not significantly affect the aldicarb sensitivity in wild type or *acr-2(lf)* background. Statistics show the comparison of *flp-1(lf); flp-18(lf)* vs *flp-1(lf); flp-18(lf) acr-2(lf)*. ***: *p*<*0.*001, **: *p*<0.01, *: *p*<0.05 by two-way ANOVA and Bonferroni post-hoc test.
(EPS)

Figure S2 Expression of *nlp-21* and *ins-22* is not affected by *acr-2(gf)*. L4 stage animals of *Punc-129::NLP-21::venus* and *Punc-129::INS-22::venus* were subjected to confocal imaging. (Top) Images from *Punc-129::NLP-21::venus* and *Punc-129::INS-22::venus* animals. Dorsal nerve cord near the bend of the gonad was imaged. NLP-21::venus was observed in coelomocyte and in the dorsal nerve cord (DNC). INS::22-venus was observed mainly in the DNC. (Middle, bottom) The fluorescence intensity was measured using ImageJ from three fluorescent patches in the coelomocyte, and the average was used for statistics. Fluorescence intensity of DNC was also examined using ImageJ. The *NLP-21::venus* expression pattern or fluorescence intensity is not affected by *acr-2(gf)*. Dashed circle indicate the position of coelomocytes. Dashed rectangles indicate the DNC region used for the measurement. Student's t-test was performed to compare fluorescence intensities. Error bars indicate SEM. Numbers in the graph indicate sample sizes.
(EPS)

Figure S3 Head neuron expression of *Pflp-18::flp-18::SL2::gfp* is not different between wild type and in *acr-2(gf).* (A) Representative confocal images of the head neurons (top) and the ventral nerve cord (bottom) in L4 animals. (B) Fluorescence intensity in a head neuron RIG is not different between wild type and *acr-2(gf)* animals. Intensity was quantified using ImageJ. Average of fluorescence intensity of the two cell bodies of RIG neuron was taken from each animal. Dashed circle in images indicates the region with two cell bodies of RIG. Numbers in the graph indicate sample sizes. Two transgenic lines (*juEx4062* and *juEx4073*) were examined and no difference was observed between the two. Results from *juEx4073* are shown.
(EPS)

Figure S4 Expression pattern of *Punc-17β::gfp*. Images of *Punc-17β::gfp* expression in the wild type (left) and *acr-2(gf)* (right) genetic background. Expression is only seen in the A and B type motor neurons.
(EPS)

Figure S5 Expression of *flp-18* by aldicarb and mecamylamine treatment. (A) *Pflp-18::flp-18::SL2::gfp* expression in the ventral nerve cord is increased by aldicarb and decreased by mecamylamine treatment. (top) Representative images of the ventral nerve cord with and without the drug treatment. White boxes indicate the region of cell body and the enlarged images of the region are shown on right of each image. (bottom) Quantification of GFP fluorescence intensity in cell body. Two transgenic lines (*juEx4062* and *juEx4073*) were examined. Results from *juEx4073* are shown. (B) Expression level of *Punc-17β::gfp* was not affected by drug treatments. GFP fluorescence intensity in cell body was quantified using ImageJ.
(EPS)

Figure S6 *ckr-2* does not affect *acr-2(gf)* convulsions. Loss of *ckr-2* or *ckr-2* in combination with *flp-18* does not affect the convulsion frequency of *acr-2(gf)*. Quantification method is the same as in others.
(EPS)

Figure S7 Loss of *flp-1* causes increased aldicarb sensitivity in *npr-5: npr-1 acr-2(gf)* background. Animals were placed on an NGM plate with 150 µM aldicarb and non-paralyzed worms were counted every 30 minutes. Loss of *flp-1* enhances aldicarb sensitivity of *npr-5; npr-1 acr-2(gf)*. Statistics show the comparison of *flp-1(lf); npr-5(lf); npr-1(lf) acr-2(gf)* vs *npr-5(lf); npr-1(lf) acr-2(gf)*. *: *p*<0.05 by two-way ANOVA and Bonferroni post-hoc test.
(EPS)

Protocol S1 Supplementary procedure for Figure S5.
(DOCX)

Table S1 Strains and Genotypes.
(DOCX)

Table S2 DNA Constructs.
(DOCX)

Video S1 Locomotion of adult *acr-2(gf)* animals.
(WMV)

Video S2 Locomotion of adult *sbt-1(lf);acr-2(gf)* animals.
(WMV)

Video S3 Locomotion of adult *unc-31(lf); acr-2(gf)* animals.
(WMV)

Video S4 Locomotion of adult *eg-3(lf); acr-2(gf)* animals.
(WMV)

Video S5 Locomotion of adult *flp-1(lf); flp-18(lf) acr-2(gf)* animals.
(WMV)
