# Supplementary material for: Neuropeptides Function in a Homeostatic Manner to Modulate Excitation-Inhibition Imbalance in C. elegans
Source: PLoS Genet. 2013 May 2;9(5):e1003472. doi: 10.1371/journal.pgen.1003472 (PMC3642046; doi:10.1371/journal.pgen.1003472)
Supplement: Protocol S1 — Supplementary procedure for Figure S5. (DOCX) [file pgen.1003472.s008.docx]

Protocol S1: Supplementary procedure for Figure S5

**Pharmacology analysis on FLP-18 expression**

For aldicarb treatment, L4 animals were picked the day before experiment. Animals were transferred to NGM plates containing 500 μM aldicarb seeded with OP50 and were subjected to confocal imaging after 6 hours. For mecamylamine treatment, young adult animals were allowed to lay eggs for 3 hours on NGM plates containing 100 μM mecamylamine seeded with OP50. Animals were kept on the mecamylamine plates for 3 days until they were in stage L4 and subjected to confocal imaging. ImageJ was used to analyze the figures. After background subtraction, GFP fluorescence intensity in the VB8 cell body was quantified.
