# Supplementary material for: Neuropeptides Function in a Homeostatic Manner to Modulate Excitation-Inhibition Imbalance in C. elegans
Source: PLoS Genet. 2013 May 2;9(5):e1003472. doi: 10.1371/journal.pgen.1003472 (PMC3642046; doi:10.1371/journal.pgen.1003472)
Supplement: Table S1 — Strains and Genotypes. (DOCX) [file pgen.1003472.s009.docx]

**Supplementary Information**

**Table S1 Strains and Genotypes**

| **Strain Number** | **Genotype** | **Allele or Transgene Information (reference)** |
| --- | --- | --- |
| CZ10402 | *acr-2(n2420)X* | *n2420:* g925a Val309Met [1] |
| CB928 | *unc-31(e928)IV* | *e928*: 5.2kb deletion [2] |
| MT6651 | *unc-31(e928)IV; acr-2(n2420)X* |  |
| CZ10347 | *unc-31(e928)IV; acr-2(n2420)X; Prgef-1::unc-31 (juEx2246)* | *Pregf-1::unc-31cDNA (pCZGY870)* - 35 ng/μl,  *Pttx-3::rfp* – 75 ng/μl |
| CZ10841 | *unc-31(e928)IV; acr-2(n2420)X; Punc-17**β::unc-31 (juEx2374)* | *Punc-17β:unc-31cDNA (KG126)* - 35 ng/μl  *[3],*  *Pttx-3:rfp* - 75 ng/μl |
| CZ10302 | *unc-31(e928)IV; acr-2(n2420)X; Punc-25::unc-31 (juEx2244)* | *Punc-25::unc-31cDNA (pCZGY868)* – 35 ng/μl,  *Pttx-3::rfp* – 75 ng/μl |
| CZ10791 | *unc-31(e928)IV; acr-2(n2420)X; Pnmr-1::unc-31 (juEx2353)* | *Pnmr-1::unc-31cDNA (pCZGY904)* – 35 ng/μl,  *Pttx-3::rfp* – 75 ng/μl |
| CZ11285 | *acr-2(n2420)X;* *Punc-17β:unc-31 (juEx2374)* |  |
| CZ10637 | *egl-21(n476)IV; acr-2(n2420)X* | *n476:* 123 bp deletion [4] |
| CZ10879 | *egl-3(nr2090)V; acr-2(n2420)X* | *nr2090:* 1188 bp deletion [5] |
| CZ11984 | *egl-21(n476)IV; egl-3(nr2090)V; acr-2(n2420)* |  |
| CZ11376 | *sbt-1(ok901)V; acr-2(n2420)X* | *ok901:* 1382 bp deletion [6] |
| CZ10346 | *unc-31(e928)IV; egl-3(n589)V;*  *acr-2(n2420)X* | *n589:* G1487a Cys496Tyr [5] |
| CZ9315 | *egl-3(n589)V; acr-2(n2420)X* |  |
| CZ10677 | *egl-3(ok979)V; acr-2(n2420)X* | *ok979:* 1579 bp deletion |
| CZ11977 | *egl-3(ok979)V; acr-2(n2420)X; Prgef-1::egl-3(juEx2720)* | *Prgef-1:egl-3 (pCZGY1076) –* 20 ng/μl,  *Pttx-3::rfp –* 75 ng/μl |
| CZ11854 | *egl-3(ok979)V; acr-2(n2420)X; Pglr-1::egl-3(juEx2668)* | *Pglr-1:egl-3 (KP509) –* 20 ng/μl [5],  *Pttx-3:rfp –* 75 ng/μl |
| CZ12111 | *egl-3(ok979)V; acr-2(n2420)X; Punc-17β::egl-3(juEx2774)* | *Punc-17β::egl-3 (pCZGY1097) –* 20 ng/μl,  *Pttx-3:rfp –* 75 ng/μl |
| CZ11750 | *egl-3(ok979)V; acr-2(n2420)X; Pacr-2::egl-3(juEx2640)* | *Pacr-2::egl-3 (KP677) –* 20 ng/μl [4], *Pttx-3::rfp –* 75 ng/μl |
| CZ9315 | *egl-3(n589)V; acr-2(n2420)X* |  |
| CZ10354 | *egl-21(n611)IV; acr-2(n2420)X* | *n611:* g1071a, Trp357Stop |
| CZ9524 | *flp-1(yn4)IV; acr-2(n2420)X* | *yn4:* 2.1kb deletion [7] |
| CZ10144 | *flp-9(ok2730)IV; acr-2(n2420)X* | *ok2730:* 432 bp deletion + 19 bp insertion |
| CZ10973 | *flp-11(tm2706)X acr-2(n2420)X* | *tm2706:* 154 bp deletion |
| CZ10927 | *flp-13(tm2427)IV; acr-2(n2420)X* | *tm2427:* 380 bp deletion |
| CZ10676 | *flp-18(tm2179)X acr-2(n2420)X* | *tm2179:* 1286 bp deletion |
| CZ10429 | *acr-2(n2420)X flp-20(ok2964)X* | *ok2964:* about 300 bp deletion |
| CZ11479 | *flp-21(ok889)V; acr-2(n2420)X* | *ok889:* 1786 bp deletion |
| CZ12583 | *acr-2(n2420)X nlp-3(tm3023)X* | *tm3023:* 354 bp deletion |
| CZ14323 | *acr-2(n2420)X nlp-7(tm2984)X* | *tm2984:* 1758 bp deletion |
| CZ12171 | *nlp-9(tm3572)V; acr-2(n2420)X* | *tm3572:* 110 bp deletion + 2 bp insertion |
| CZ10040 | *nlp-12(ok335)IV; acr-2(n2420)X* | *ok335:* 1070 bp deletion |
| CZ12610 | *acr-2(n2420)X nlp-14(tm1880)X* | *tm1880:* 661 bp deletion + 12 bp insertion |
| CZ12286 | *nlp-15(ok1512)I; acr-2(n2420)X* | *ok1512:* 889 bp deletion |
| CZ10948 | *ins-3(ok2488)II; acr-2(n2420)X* | *ok2488:* 1449 bp deletion |
| CZ10857 | *ins-4(ok3534)II; acr-2(n2420)X* | *ok3534:* about 400bp deletion |
| CZ10877 | *ins-11(tm1053)II; acr-2(n2420)X* | *tm1053:* 341 bp deletion |
| CZ11427 | *ins-15(ok3444)II; acr-2(n2420)X* | *ok3444:* about 600bp deletion |
| CZ10774 | *ins-18(ok1672)I; acr-2(n2420)X* | *ok1672*: 940 bp |
| CZ11414 | *ins-22(ok3616)III; acr-2(n2420)X* | *ok3616:* about 700 bp deletion |
| CZ11768 | *ins-27(ok2474)I; acr-2(n2420)X* | *ok2474:* 461 bp deletion |
| CZ11480 | *ins-28(ok2722)I; acr-2(n2420)X* | *ok2722:* 584 bp deletion |
| CZ11481 | *ins-30(ok2343)I; acr-2(n2420)X* | *ok2343:* 1078 bp deletion |
| CZ11325 | *ins-35(ok3297)V; acr-2(n2420)X* | *ok3297:* about 600 bp deletion |
| CZ11341 | *flp-1(yn4)IV; flp-18(tm2179)X*  *acr-2(n2420)X* |  |
| CZ14895 | *flp-9(ok2730)IV; flp-18(tm2179)X acr-2(n2420)X* |  |
| CZ14936 | *flp-13(tm2427)IV; flp-18(tm2179)X acr-2(n2420)X* |  |
| CZ14915 | *flp-21(ok889)V;* *flp-18(tm2179) acr-2(n2420)X* |  |
| CZ15084 | *nlp-9(tm3572)V; flp-18(tm2179) acr-2(n2420)X* |  |
| CZ14680 | *nlp-12(ok335)IV; flp-18(tm2179) acr-2(n2420)X* |  |
| CZ14682 | *ins-22(ok3616)III; flp-18(tm2179) acr-2(n2420)X* |  |
| CZ14934 | *flp-18(db99)X acr-2(n2420)* | *db99:* 2016 bp deletion [8] |
| CZ15458 | *flp-1(yn4)IV; flp-18(db99)X*  *acr-2(n2420)X* |  |
| NY16 | *flp-1(yn4)IV* |  |
| CZ10206 | *flp-18(tm2179)X* |  |
| CZ12078 | *flp-1(yn4)IV; flp-18(tm2179)X* |  |
| CZ15048 | *Pflp-18::flp-18::SL2::gfp (juEx4073)* | *Pflp-18::flp-18::SL2::gfp –* 5 ng/μl [8],  *Pttx-3::rfp –* 75 ng/μl |
| CZ15032 | *Pflp-18::flp-18::SL2::gfp(juEx4062)* | *Pflp-18::flp-18::SL2::gfp –* 5 ng/μl [8],  *Pttx-3::rfp –* 75 ng/μl |
| CZ15164 | *acr-2(n2420)X; Pflp-18::flp-18::SL2::gfp (juEx4073)* |  |
| CZ15859 | *acr-2(n2420)X; Pflp-18::flp-18::SL2::gfp (juEx4062)* | *Pflp-18::flp-18::SL2::gfp –* 5 ng/μl [8],  *Pttx-3::rfp –* 75 ng/μl |
| CZ15509 | *flp-1(yn4)IV; flp-18(tm2179)*  *acr-2(n2420)X;Pregf-1::flp-1 (juEx4302)* | *Prgef-1::flp-1(pCZGY1692)* -5ng/μl,  *Pnmr-1::mCherry*-20ng/μl |
| CZ15510 | *flp-1(yn4)IV; flp-18(tm2179)*  *acr-2(n2420)X;Prgef-1:flp-1*  *(juEx4303)* | *Prgef-1::flp-1(pCZGY1692)*-5ng/μl,  *Pnmr-1::mCherry* - 20ng/μl |
| CZ16375 | *acr-2(n2420)X; Pflp-18:flp-18-SL2-gfp(juEx4073); Pacr-5:mCherry (juEx3226)* | *Pacr-5::mCherry –* 20ng/μl, *Pttx-3::gfp* - 50ng/μl |
| CZ16377 | *acr-2(n2420)X; Pflp-18:flp-18::SL2::gfp(juEx4073); Punc-4:mCherry (juEx3292)* | *Punc-4::mCherry –* 20ng/μl, *Pttx3::gfp* – 50ng/μl |
| CZ16473 | *acr-2(n2420)X; Pttr-39::mCherry(juIs223); Pflp-18::flp-18::SL2-gfp(juEx4073)* |  |
| CZ16466 | *egl-3(nr2090)V sbt-1(ok901)V; acr-2(n2420)X* |  |
| CZ10432 | *npr-1(ok1447)X acr-2(n2420)X* | *ok1447:* 1263bp deletion |
| CZ15107 | *flp-1(yn4)IV; npr-1(ok1447)X*  *acr-2(n2420)X* |  |
| CZ9713 | *npr-5(ok1583)V; acr-2(n2420)X* | *ok1583:* 784bp deletion |
| CZ14381 | *flp-1(yn4)IV; npr-5(ok1583)V;*  *acr-2(n2420)X* |  |
| CZ15511 | *acr-2(n2420)X npr-4(tm1782)X* | *tm1782:* 1226bp deletion |
| CZ16964 | *flp-1(yn4)IV; acr-2(n2420)X*  *npr-4(tm1782)X* |  |
| CZ17281 | *npr-5(ok1583)V; acr-2(n2420)X npr-4(tm1782)X* |  |
| CZ17437 | *npr-5(ok1583); npr-1(ok1447)X acr-2(n2420)X* |  |
| CZ17438 | *egl-21(tm5578)IV; acr-2(n2420)* | *tm5578:* 839bp deletion + 24bp insertion |
| CZ17439 | *flp-1(yn4)IV; npr-5(ok1583)V; acr-2(n2420)X npr-4(tm1782)X* |  |
| CZ17215 | *egl-21(n476)IV; flp-18(tm2179)X acr-2(n2420)X* |  |
| CZ17214 | *acr-2(n2420)X; Pflp-18::flp-18::SL2:gfp* |  |
| AX1444 | *Pflp-18:flp-18::SL2::gfp (dbIs1)* | [8] |
| CZ18094 | *npr-5(ok1583)V; acr-2(n2420)X; Pflp-18::flp-18::SL2::gfp(dbIs1)* |  |
| CZ18095 | *npr-1(ok1447)X acr-2(n2420)X; Pflp-18::flp-18::SL2::gfp(dbIs1)* |  |
| CZ18096 | *npr-5(ok1583)V; acr-2(n2420)X npr-4(tm1782)X; Pflp-18::flp-18::SL2::gfp(dbIs1)* |  |
| CZ18097 | *npr-5(ok1583)V; npr-1(ok1447)X acr-2(n2420)X; Pflp-18::flp-18::SL2::gfp(dbIs1)* |  |
| CZ18098 | *flp-1(yn4)IV; flp-18(tm2179)X acr-2(n2420)X; Pflp-18::flp-18::SL2::gfp(dbIs1)* |  |
| CZ18099 | *npr-1(ok1447)X acr-2(n2420)X; Prgef-1::flp-1(juEx4303)* |  |
| CZ18100 | *npr-5(ok1583)V; npr-1(ok1447)X acr-2(n2420)X; Prgef-1::flp-1(juEx4303)* |  |
| CZ18156 | *npr-5(ok1583)V; acr-2(n2420)X npr-4(tm1782)X; Prgef-1::flp-1(juEx4303)* |  |
| CZ18157 | *npr-5(ok1583)V; acr-2(n2420)X; Prgef-1::flp-1(juEx4303)* |  |
| CZ18304 | *npr-5(ok1583)V; npr-1(ok1447)X acr-2(n2420)X; Punc-25::npr-1(juEx5423)* | *Punc-25::npr-1::gfp(KP1856)* *–* 60ng/μl, *Pmec4::rfp –* 100ng/μl |
| CZ18305 | *npr-5(ok1583)V; npr-1(ok1447)X acr-2(n2420)X; Punc-25::npr-1(juEx5424)* | *Punc-25::npr-1::gfp(KP1856)* *–* 60ng/μl, *Pmec4::rfp –* 100ng/μl |
| CZ18306 | *npr-5(ok1583)V; npr-1(ok1447)X acr-2(n2420)X; Punc-25::npr-1(juEx5425)* | *Punc-25::npr-1::gfp(KP1856)* *–* 60ng/μl, *Pmec4::rfp –* 100ng/μl |
| CZ18303 | *npr-5(ok1583)V; npr-1(ok1447)X acr-2(n2420)X; Pnpr-5::npr-5(dbEx2)* | *Pnpr-5::npr-5* [8] |
| CZ18313 | *npr-5(ok1583)V; npr-1(ok1447)X acr-2(n2420)X; Pmyo-3::npr-5(juEx5468)* | *Pmyo-3::npr-5*  *(PCZGY2197) –*50ng/μl, *Pmec4::rfp –* 65ng/μl |
| CZ18314 | *npr-5(ok1583)V; npr-1(ok1447)X acr-2(n2420)X; Pmyo-3::npr-5(juEx5469)* | *Pmyo-3::npr-5*  *(PCZGY2197) –*50ng/μl, *Pmec4::rfp –* 65ng/μ |
| CZ18183 | *ckr-2(**tm3082)III; acr-2(n2420)X* | *tm3082*: 569 bp deletion |
| CZ18184 | *ckr-2(tm3082)III; flp-18(tm2179)X acr-2(n2420)X* |  |
| CZ12117 | *Punc-17β::gfp(juEx2777)* | *Punc-17β::gfp*  *(PCZGY1098)* |
| CZ17400 | *acr-2(n2420)X; Punc-17β::gfp(juEx2777)* |  |
| CZ18559 | *flp-1(yn4)IV; npr-5(ok1588)V;npr-1(ok1447)X acr-2(n2420)X* |  |
| CZ9677 | *acr-2(ok1887)X* | *ok1887:* 2857 bp deletion and 420 bp insertion[1] |
| CZ18101 | *flp-1(yn4(IV);flp-18(tm2179)X acr-2(ok1887)X* |  |
| CZ9267 | *Punc-129::nlp-21::venus(nuIs183)* | [9] |
| CZ9317 | *acr-2(n2420)X; Punc-129::nlp-21::venus(nuIs183)* |  |
| CZ9266 | *Punc-129::ins-22::venus(nuIs195)* | [10] |
| CZ9318 | *acr-2(n2420)X; Punc-129::ins-22::venus(nuIs195)* |  |

Information on tm and ok alleles came from [www.wormbase.org](http://www.wormbase.org) and www.cbs.umn.edu/CGC/

1. Jospin M, Qi YB, Stawicki TM, Boulin T, Schuske KR, et al. (2009) A neuronal acetylcholine receptor regulates the balance of muscle excitation and inhibition in Caenorhabditis elegans. PLoS Biol 7: e1000265.

2. Livingstone D (1991) Studies on the unc-31 gene of Caenorhabditis elegans: University of Cambridge.

3. Charlie NK, Schade MA, Thomure AM, Miller KG (2006) Presynaptic UNC-31 (CAPS) is required to activate the G alpha(s) pathway of the Caenorhabditis elegans synaptic signaling network. Genetics 172: 943-961.

4. Jacob TC, Kaplan JM (2003) The EGL-21 carboxypeptidase E facilitates acetylcholine release at Caenorhabditis elegans neuromuscular junctions. J Neurosci 23: 2122-2130.

5. Kass J, Jacob TC, Kim P, Kaplan JM (2001) The EGL-3 proprotein convertase regulates mechanosensory responses of Caenorhabditis elegans. J Neurosci 21: 9265-9272.

6. Husson SJ, Schoofs L (2007) Altered neuropeptide profile of Caenorhabditis elegans lacking the chaperone protein 7B2 as analyzed by mass spectrometry. FEBS Lett 581: 4288-4292.

7. Nelson LS, Rosoff ML, Li C (1998) Disruption of a neuropeptide gene, flp-1, causes multiple behavioral defects in Caenorhabditis elegans. Science 281: 1686-1690.

8. Cohen M, Reale V, Olofsson B, Knights A, Evans P, et al. (2009) Coordinated regulation of foraging and metabolism in C. elegans by RFamide neuropeptide signaling. Cell Metab 9: 375-385.

9. Sieburth D, Madison JM, Kaplan JM (2007) PKC-1 regulates secretion of neuropeptides. Nat Neurosci 10: 49-57.

10. Sieburth D, Ch'ng Q, Dybbs M, Tavazoie M, Kennedy S, et al. (2005) Systematic analysis of genes required for synapse structure and function. Nature 436: 510-517.
