# Supplementary material for: Neuropeptides Function in a Homeostatic Manner to Modulate Excitation-Inhibition Imbalance in C. elegans
Source: PLoS Genet. 2013 May 2;9(5):e1003472. doi: 10.1371/journal.pgen.1003472 (PMC3642046; doi:10.1371/journal.pgen.1003472)
Supplement: Table S2 — DNA Constructs. (DOCX) [file pgen.1003472.s010.docx]

**Table S2. DNA Constructs**

| **Plasmid *** | **Promoter** | **Gene** |
| --- | --- | --- |
| *Prgef-1::egl-3*  *(PCZGY1076)* | 3.5kb upstream of ATG of *rgef-1*[9] | 4.0kb *egl-3* genomic DNA fragment amplified using the following primers :YJ7092  5’-atgaaaaacacacatgtcgacc-3’  YJ7093 5’-ttagtggctgcgtttgtggg-3’ |
| *Prgef-1::unc-31*  *(PCZGY870)* | 3.5kb upstream of ATG of *rgef-1*[9] | *unc-31* cDNA[10] |
| *Punc-25::unc-31*  *(PCZGY868)* | 1.3kb upstream of ATG of *unc-*25 | *unc-31* cDNA[10] |
| *Pnmr-1::unc-31*  *(PCZGY904)* | 1.1kb upstream of ATG of *nmr-1* | *unc-31* cDNA[10] |
| *Punc-17β::egl-3*  *(PCZGY1097)* | 0.5kb upstream of ATG of *unc-17β*[3] | *egl-3* genomic DNA, same as *PCXGY1076* |
| *Prgef-1::flp-1*  *(PCZGY1692)* | 3.5kb upstream of ATG of *rgef-1*[9] | 1.3kb *flp-1* genomic DNA fragment amplified using the following primers:  YJ8345  5’- atgactctgctctaccaagtagg-3’  YJ8346  5’- ttattttccgaaacgaaggaaatttg-3’ |
| *Pmyo-3::npr-5*  *(PCZGY2197)* | 2.4kb upstream of ATG of *myo-3*[11] | *npr-5* cDNA[8] |
| *Punc-17β::gfp*  *(PCZGY1098)* | 0.5kb upstream of ATG of *unc-17β*[3] | GFP cDNA |

* DNA constructs were generated using Gateway Cloning Technology (Invitrogen, CA).
